# Stepping outside the consultation room. On nurse–patient relationships and nursing responsibilities during a type 2 diabetes walking project

**DOI:** 10.1111/jan.14037

**Published:** 2019-06-06

**Authors:** Mirjam Stuij, Agnes Elling‐Machartzki, Tineke A. Abma

**Affiliations:** ^1^ Department of Medical Humanities, Amsterdam Public Health Research Institute Amsterdam UMC (location VUmc) Amsterdam the Netherlands; ^2^ Mulier Institute Utrecht the Netherlands

**Keywords:** care practices, ethnography, nurse‐led intervention, nurse–patient relationships, nursing, nursing responsibilities, physical activity, shared care, system and lifeworld dynamics, type 2 diabetes

## Abstract

**Aims:**

To examine the care practices of nurses during the organization of 20 weeks of walking sessions for people with type 2 diabetes and to reflect on implications for nurse–patient relationships and nursing responsibilities in the provision of physical activity (PA) care.

**Design:**

Qualitative, ethnographic study.

**Methods:**

Almost 70 hr of field work was completed by participant observations and informal conversations with nurses and participating patients of two different walking groups (April–October 2016). Analysis of field notes followed an inductive holistic‐content approach, using both within‐case and across‐case analysis.

**Results:**

The analysis revealed four main themes related to the nurses’ care practices: (a) organizational efforts; (b) combining group and individual care; (c) stepping in‐ and outside the patient mode; and (d) implications back inside the consultation room. Underlying these themes was a process of relational development, both with and among patients.

**Conclusion:**

Stepping outside the consultation room seems to offer more space for patients’ lifeworld narratives and contribute to more continuous and person‐centred care. However, it also raises new questions about the provision of PA care and nursing responsibilities in this.

**Impact:**

Current nursing repertoires for PA counselling in type 2 diabetes care are insufficient and might be extended by organizing walking sessions for patients. Related nursing care practices impacted relationships both with and among participating patients. These have consequences for boundaries of both nursing responsibilities and care provision.

## Abstract (zh)

**目的:**

研究护士在为2型糖尿病患者组织为期20周的步行项目期间的护理实践,并反思在提供身体活动(PA)护理中对护患关系和护理职责的影响。

**设计:**

定性、人类学研究。

**方法:**

通过参与者观察和与两个不同步行组的护士和参与患者的非正式谈话(2016年4月至10月),完成了近70小时的现场工作。现场笔记的分析遵循归纳的整体内容方法,使用案例内和跨案例分析。

**结果:**

分析揭示了与护士护理实践相关的四个主要主题:(a)组织工作;(b)将集体和个人护理结合起来;(c)走入和走出患者模式;以及(d)回到诊察室的启示。在这些主题的基础上是一个关系发展的过程,无论是与患者之间还是与患者之间。

**结论:**

走出诊察室似乎为患者的生活世界叙述提供了更多的空间,并有助于更加持续和以人为中心的护理。然而,这也提出了关于提供身体活动护理和护理责任的新问题。

**影响:**

目前2型糖尿病护理中身体活动咨询的护理计划不足,可以通过为患者组织步行项目来延长。相关的护理实践影响了和参与患者以及患者之间的关系。这些都会影响到护理责任和护理规定的界限。

## 1 INTRODUCTION

Worldwide, physical activity (PA) recommendations are part of clinical practice guidelines for the management of type 2 diabetes (IDF, 2017). However, previous studies indicate that PA is not an easy topic in diabetes care. While healthcare professionals consider it to be important, they mention uncertainty about the effectiveness of their counselling and uncomfortable feelings about providing detailed advices (Hébert, Caughy, & Shuval, 2012). Their main repertoire inside the consultation room consists of education, offering advices and encouragement, or referral to exercise specialists, but this seems insufficient to support patients to adopt more active lifestyles (IDF, 2017; Stuij, 2018).

To extent the professional 'PA care' repertoire, a Dutch project was initiated to facilitate healthcare professionals in the organization of weekly walking sessions for patients with type 2 diabetes. This was scheduled for 20 weeks with participation in a 4 Days Walking Event as a shared goal. After small initiatives in 2014 and 2015, these walking sessions were organized as part of a yearly 'National Diabetes Challenge' (NDC) on 125 locations in 2016 and over 200 locations in 2018 (Bas van de Goor Foundation, 2016, 2018).

Many of these organizing professionals are nurses, as they offer most of the practical type 2 diabetes care in the Netherlands. Over 85% of Dutch people with type 2 diabetes is treated in primary care (Vektis, 2015), where general practitioners have the final responsibility, but generally most care is handed over to general practice nurses (*praktijkondersteuners somatiek*). These are registered nurses who, for instance, educate patients about diabetes and lifestyle (Freund et al., 2015). Patients in need of more complex care are referred to an internist in secondary care, where nurses specialized in diabetes can offer most of the practical care (Houweling et al., 2009).

Several studies point towards the potential of nurse‐led interventions aimed at healthy lifestyle and chronic disease management (Sargent, Forrest, & Parker, 2012; Stephen, McInnes, & Halcomb, 2018). However, these might change existing practices and ask for professional, organizational and policy adaptations accordingly and challenge professional responsibilities (Stephen et al., 2018). Therefore, the aim of this study is to examine the care practices of nurses during the organization of walking sessions and to reflect on implications for nurse–patient relationships and nursing responsibilities in the provision of PA care.

### 1.1 Background

Review studies show competing priorities and lack of time, training and reimbursement as the most important barriers to PA promotion practices for healthcare professionals, while good relationships with patients, knowledge about their personal lives and active lifestyles of professionals themselves are mentioned to be enhancing (Hébert et al., 2012; Huijg et al., 2015). Qualitative insights indicate ambivalent feelings of professionals about the understanding of inactive patient behaviour and regarding professional views related to individual and professional responsibilities for behaviour change (Stuij, 2018). These findings illustrate the importance of the care context and the quality of relationships with patients, as well as the existence of morally ambivalent tensions in the provision of PA care (cf. Strandås & Bondas, 2018; Wiechula et al., 2016).

Overall, nurses seem to be mostly medically oriented, while patients experience little attention to personal, social and practical challenges to integrate illness into their daily life (Been‐Dahmen, Dwarswaard, Hazes, Van Staa, & Ista, 2015; cf. Brundisini, Vanstone, Hulan, DeJean, & Giacomini, 2015). This points towards a gap between system and lifeworld, proposed as a distinction between a scientific, bureaucratic and impersonal logic on the one hand and a logic based on values and narratively embedded face‐to‐face relations on the other hand (Habermas, 1984; Kunneman, 2015; Van den Ende & Kunneman, 2008). This distinction is considered a structural feature of care contexts, resulting in an ongoing tension. The course of a consultation, for instance, can be guided by blood sample results or the question 'how are you?'.

Rather than dealing with a structural opposition between these logics, healthcare professionals can also operate in an in‐between domain where both system and lifeworld are important; it depends on the situation which one prevails (Abma, Leyerzapf, & Landeweer, 2017; Kunneman, 2015). Furthermore, it is argued that both logics generate morally ambivalent dilemma's, for instance, related to solidarity, division of services and personal interests (Van den Ende & Kunneman, 2008). Therefore, professional care practices always involve moral choices about the provision and organization of ‘good’ care.

Against this background, the NDC provides an interesting extension of the PA care repertoire, as nurses actually go walking with their patients instead of giving advices or a referral. It is initiated by the *Bas van de Goor Foundation*, a small non‐profit organization that promotes sport and PA for people with diabetes and aims to lower the threshold for patients to become more active. During the NDC, the foundation facilitates nurses in the organization of weekly walking sessions; for instance, they provide communication materials and a project plan with training schedules, have a helpdesk and organize a final collective walking event (Bas van de Goor Foundation, 2015).

Several features of the nurses’ care practices in this project are different from their regular practices. Overall, these new practices seem to be more outside the system world and into the patients’ lifeworld than regular care practices. First, care is provided in a different context, because nurses and patients literally step outside the consultation room to walk outdoors. Second, instead of one‐to‐one care, nurses provide care to a group of people. These two features might impact nurse–patient relationships (Wiechula et al., 2016) and offer possibilities for patients to share experiences and learn from each other (Oftedal, Karlsen, & Bru, 2010). Third, it is a bottom‐up project without protocols about how to arrange care as only facilitation is offered and the organization is left open to the nurses themselves—offering space for learning and adaptation along the way (Horstman & Houtepen, 2008).

Therefore, it is expected that these new care practices create opportunities to better meet the caring needs of patients, but also raise new moral questions about the provision and organization of care. A study of the learning process and professional development of the organizing nurses might offer a starting point for a reflection on nurse–patient relationships and nursing responsibilities in the provision of PA care.

## 2 THE STUDY

### 2.1 Aims

The aim of the study was to examine the care practices of nurses during the organization of 20 weeks of walking sessions for people with type 2 diabetes and to reflect on implications for nurse–patient relationships and nursing responsibilities in the provision of PA care.

### 2.2 Design

This study was conducted from a social constructivist paradigm: we approached reality as multiple and socially constructed through lived experiences and interactions (Creswell & Poth, 2018). To examine the care practices carefully, an ethnographic study was carried out where the researcher (first author) participated in two walking groups. A comparison of two different locations enabled us to better understand these practices, as differences and similarities as well as specificities of the context emerged (Ayres, Kavanaugh, & Knafl, 2003). Furthermore, as we did not want to limit our observations, we did not define care practices in advance. Instead, we observed what was actually done in practice (Mol, Moser, & Pols, 2010) what the nurses and others told the researcher and each other about it.

### 2.3 Participants

To understand what was new compared with regular care practices, the nurses in this study needed to take part in the project for the first time. Further inclusion criteria were pragmatic, namely feasibility for the researcher—e.g. walks not scheduled at the same time—and willingness of the nurses. One nurse, henceforth 'Ellen', presented herself after a presentation of the researcher at a general information meeting about the NDC. The second nurse, 'Nicole', was included after an e‐mail sent by the *Bas van de Goor Foundation*. Relevant characteristics are listed in Table 1.

**Table 1:** Details of the organizing nurses

Pseudonym	'Nicole' (location N)	'Ellen' (location E)
Position	Diabetes nurse	General practice nurse (*praktijkondersteuner somatiek* in Dutch)
Employed at	Hospital (secondary care)	Healthcare centre (primary care, a centre where general practitioners and several other (paramedic) healthcare professionals are located)
Gender	Female	Female
Years of working experience	Over 20 of which 15 years as diabetes nurse	12
Experiences with walking groups before	None	Already facilitated a small weekly walking group with her colleague for a few years

### 2.4 Data collection

The researcher was educated as a medical sociologist and has 10 years of experience in qualitative research, including participant observations, with a focus on type 2 diabetes care and PA for the past 5 years. She participated in two walking groups from the onset until the final event and meetings (April–October 2016). She held informal conversations with the nurses and patients and made observations, both written down in field notes afterwards. In total, almost 70 hr of field work was completed.

As the researcher did not know the research setting beforehand, field notes were collected in an unstructured way, without specific questions or observation protocols (Hammersley & Atkinson, 2007). They were written in both first and third person. This was not a conscious decision, but reflected the researcher's presence as 'the lens through which one sees' as well as someone depicting others (Emerson, Fretz, & Shaw, 2011:101). As she was present from the beginning of the project onwards, she became part of the groups, in an undefined role mingling between observer, participating walker and scheduled as a walking guide at times. This enabled her to relate an 'insider' participant view to an 'outsider' academic view to make sense of the nurses’ care practices (Green & Thorogood, 2018). She reflected on this in reflexive field notes.

### 2.5 Ethical considerations

This study was conducted as part of a larger project, which was waived requirement of full medical ethical approval by the university's medical research ethics committee and further discussed with its privacy officer. As a result, the organizing nurses were informed by a letter explaining the aims, practical implications and benefits of the study and efforts to ensure confidentiality. This was talked about with each nurse and put down in an informed consent form. This form also ensured openness of the researcher about the study towards the participating patients to obtain oral informed consent. In practice, she introduced herself to the groups during the information meeting and first walk and then whenever necessary to ensure that everyone was informed. For privacy reasons, minimum information about the locations and people involved is provided.

### 2.6 Data analysis

Field notes were analysed using MaxQDA Version 12, following an inductive holistic–content approach (Lieblich, Tuval‐Mashiach, & Zilber, 1998): we interpreted practices in the context of other observations to connect these to each other, locate them in time and place and thereby explore the process and development of the nurses during the project (Lieblich et al., 1998; cf. Beal, 2013). We altered between (re‐)reading field notes, open coding and further arrangements of codes (Elo & Kyngäs, 2008), which resulted in four major closely connected and more abstract themes. Moving between within‐case and across‐case analysis sensitized us towards critical reflections on these codes and themes and the preservation of the contextual richness of each case (Ayres et al., 2003). The analysis was carried out by the first author and frequently discussed with the others—a critical sport scholar and an interdisciplinary scholar in the fields of social sciences, humanities and medical sciences with a background in nursing.

### 2.7 Rigour

To enhance rigour, we used the following strategies (Polit & Beck, 2012): (a) prolonged engagement and persistent observation in the field by being present for the whole project; (b) space triangulation by collecting data on two locations; (c) informal member checks by discussion of the findings with nurses and patients during the walks and meetings; (d) reflexive journaling during data collection and analysis and in‐depth discussions about this in the author team; and (e) thick description by providing rich and vivid descriptions.

## 3 FINDINGS

The analysis resulted in four main themes related to the care practices of the nurses: (a) organizational efforts; (b) combining group and individual care; (c) stepping in‐ and outside the patient mode; and (d) implications back inside the consultation room. These themes were closely connected to each other; together they describe the context and process, especially related to developments of relations with and among patients.

### 3.1 Organizational efforts

Both nurses started with about 30 patients; included through consultations, information meetings and leaflets. These were not all their own patients, but also other people with type 2 diabetes living in the surrounding. Indeed, most in Ellen's group was not her patient. This indicates the nurses’ responsibilities went beyond their own patient files from the beginning onwards.

The project demanded relatively considerable organizational efforts of the nurses. These comprised the practical work necessary for the realization of the walks. Most of these efforts were calculated in beforehand, although the amount of necessary time was unforeseen, like the actual walking, tasks related to the recruitment of participating patients and 'walking guides', the planning of routes and the arrangement of devices and sweets to measure and, when necessary, increase blood glucose levels along the way. However, the nurses also needed to be flexible with respect to uncertain conditions, like the weather and number of people actually present during a walk. Although these efforts were necessary for both groups, Ellen's earlier experiences with a walking group seemed to help her. Especially the planning of routes seemed to cause less stress, as she already knew possible walking routes in the surrounding quite well.

Overall, three main organizational efforts were found: (a) organizing support from colleagues; (b) personal investments; and (c) organizing follow‐up.

#### 3.1.1 Organizing support from colleagues

Both nurses made the decision to join the project themselves, because they were interested in this new approach. They got permission to do this and both were provided with about the same amount of time for the organization. Moreover, they experienced most colleagues to be positive when they told them about this project. However, they equally struggled to find practical support from colleagues. Both had one or two colleagues helping them frequently and also found some others willing and able to guide a walk now and then. But this was not something easy and seemed to cause extra stress, especially because the nurses themselves could not be there every walk, for instance, because of other professional duties or their own summer holidays. This was illustrated by the inclusion of the researcher in the guidance schedule at location E.

For Nicole, especially the inclusion of doctors turned out to be difficult, despite the efforts she took to realize this:

A lady asks why the walks are scheduled around diner time, because that is impractical with diabetes. Nicole tells her that it is more easy to empty the agenda of the internists at the end of the day. She does not tell about her own consultations. [location N, instruction meeting]

Nicole tells the group she won't be there the next four weeks and still needs to fill the schedule with guides. But she will arrange for the doctors to be there, she says. The group thinks this won't work because the doctors always have excuses: too many consultations, meetings, or whatever. [location N, walk 8]

At the end of the project, Nicole said she was disappointed about the absence of the doctors. She thought they did not dare to walk along with their patients. This probably illustrates the dominance of the system world with a strict hierarchy between doctors and patients, something the patients seemed aware of. The doctors of the other location did not join either, something Ellen also noticed and tried to change, although it seemed to be less frustrating for her. This might indicate that the patients and nurse in the hospital experienced a larger distinction between the system—and related hierarchical positions—and lifeworld than those involved with the healthcare centre.

#### 3.1.2 Personal investments

The organizational efforts were not all visible to others, as they were carried out whenever there was a spare moment. Moreover, these required the investment of the nurses’ spare time, because working hours were not sufficient. Therefore, the project entered the lifeworld of the nurses themselves. The walks of Ellen, for instance, were scheduled in the evenings. At the other location, the walks were scheduled at the end of the working day with the first half planned inside 'consultation hours'. But this also meant Nicole often seemed to be in a hurry to be there in time for the walks, illustrating a tension with regular care practices.

#### 3.1.3 Organizing follow‐up

The third main organizational effort had to do with follow‐up. These were extra efforts the nurses went into, as they were not necessary for the project. First, part of the patients quit during the project. For instance, Ellen noticed that her 'slow group had disappeared' the second walk. Indeed, at both locations, the number of patients almost halved after the first walks. During the remainder of the project, some others quit as well. Both nurses called after them to convince them to continue participation and if they did not want to, to refer them to an exercise expert. Second, the nurses cared about follow‐up after the project, to enable patients to continue to be active. Both searched in the neighbourhood for options, tried to create a social network with exercise coaches or the local walking club and organized a final meeting to inform the patients.

### 3.2 Combining individual and group care

The provision of care to a group of people was a new practice and brought along the intricacy of attentiveness towards individual needs, while, at the same time, having an eye for everyone. This was especially visible in the subthemes ‘dealing with private matters’ and ‘taking care of both quick and slow walkers’. However, the group also provided opportunities for ‘shared care’, the third subtheme.

#### 3.2.1 Dealing with private matters

Providing group care brought along issues about dealing with private matters. For instance, Nicole's group took part in a study on the effectiveness of the project and this required measurements beforehand:

The scales are taken out. Nicole weighs everyone, measures their belly and waist circumference and notes the values down on a small piece of paper. This results in some funny remarks: ‘Hey, don't hold your stomach!’, or ‘Plus ten kilos!’. Most people do not know each other, this seems to evoke uncomfortable feelings. [location N, instruction meeting]

Here, the emphasis of the system world on measurements interfered with the intimacies from the lifeworld, which are normally discussed in the privacy of the consultation room. These issues were especially faced at the beginning of the project and sorted out along the way, because ‘private’ moments were created during the walks and the nurses got used to these matters. Both showed flexibility to handle these, illustrated by remarks like ‘come by and see me tomorrow’ and ‘you can come in whenever my [consultation room] door is open’.

#### 3.2.2 Taking care of both quick and slow walkers

From the first walk onwards, patients differed in their pace because of their different conditions. This resulted in ‘quick’ and ‘slow’ walkers, something the nurses had to adapt to. For example, one patient at location N was clearly slower than all others. After the first walk, he said he needed more pauses along the way. Therefore, Nicole took along a wheelchair for the second walk, which had implications for the others as well:

Nicole is still not back [at the meeting point at the end of the walk] and the others are in doubt whether to go home or not. But then she arrives, together with her friend [a volunteer guide] and the man in the wheelchair. Nicole pushes the wheelchair and the three of them were behind the whole route. The other patients tell her they want someone walking with them in front as well, to guide them. They seem a bit annoyed. [location N, walk 2]

Nicole walked behind with this man for four walks, until he decided to quit because he could not carry on anymore. At the end of the project, Nicole indicated that she found it difficult to pay attention to everyone, especially because some demanded more than others.

Ellen used a different approach from the first walk onwards:

The leading group is waiting beneath the crossover. Ellen says she wants everyone together to see how it goes. The very last walkers are still quite a way behind and Ellen suggests we walk back and get them. ‘Vacuuming’, she calls it. As not everyone sees the benefit, we go back with a small group. [location E, walk 1]

This was an approach she used throughout the project and matched her own sport biography; Ellen used to be a running coach and participated in marathons. Elements of performance were visible in her approach, as she measured and stressed the distance and number of steps for each walk.

These two approaches illustrate differences in the organization of both nurses. This was probably dependent on many different factors—like the context, their patients with different conditions and care needs, their own preferences and Ellen's previous experiences with a walking group—and exemplifies the personal interpretation of both nurses in the provision of care. This interpretation was, many times, also a matter of improvisation because the context or needs of patients required last‐minute adaptations. As the approach of Nicole illustrated, this also raised dilemmas.

#### 3.2.3 Shared care

In the course of the project, patients also came to take care of each other. This ‘shared care’ was both practical and social and took some work off the nurses. For example, patients helped them to map out routes or distribute water bottles. Furthermore, they shared a lot of experiences and tips with each other, for instance, about what to do with high blood sugar levels, how to deal with insurance demands and where to buy good walking shoes.

Another form of shared care was a kind of social discipline that emerged: patients asked others about their absence and checked if everything was fine. In one of the groups, this social discipline continued after the project:

Ellen's group is sharing phone numbers. They're starting a WhatsApp group and agree on walking every Saturday morning for two hours. (…) Ellen says she also joined the group, ‘to keep an eye on them’, she jokes. [location E, final meeting]

For these patients, shared care resulted in a self‐organized follow‐up of the project. It illustrates a shift in the responsibility of the nurse in organizing PA care towards a delegation towards the patients themselves.

### 3.3 Stepping in‐ and outside the patient mode

Over the course of the project, relations between the nurses and patients changed. This was made explicit by one of the nurses near the end of the project:

The leading group is waiting ahead. Ellen proposes a route to continue and tells the group she really has to leave [because of a scheduled consultation]. Up to three times she says: ‘I need to get back into the patient mode now!’. [location E, walk 20]

This remark implied another mode during the walks and another attitude towards the patients. At the other location, there were also hints towards being in a different mode:

Nicole mapped out a route this time. She jokes we can walk by the ice‐cream vendor and buy an ice‐cream. (…) Indeed, we do and Nicole treats us. Some people pass, others pick the sugar free strawberry flavour. Her colleague does not want to, she seems to disagree. [location N, walk 8]

An ice‐cream was something Nicole probably would not recommend inside the consultation room and it implied her being more outside the professional mode during the walks.

Related to this, the walks offered nurses and patients an opportunity to get to know each other beyond the biomedical. For instance, many informal talks about the surroundings resulted in personal talks about where one lived or daily pursuits. Furthermore, conversations were continued over the weeks and people talked about events in between the walks. In this process of getting acquainted, both nurses also exchanged personal information about themselves, something they told they hardly did before:

Nicole wears a step counter as well. (…) She tells me she also joined the online community [they have] to make her data visible for everyone. She also added her body weight, so others can see it. She wants to lose some and she thought she'd better be fair about it in the group. [location N, walk 1]

This illustrates both an identification with patient issues and an example of shared care. Moreover, it indicates a shift where the nurse's lifeworld became part of the nurse–patient relations.

### 3.4 Implications back inside the consultation room

Near the end, both nurses narrated they noticed the conversations inside the consultation room changed; they experienced their patients to feel more free to ask questions and tell certain things, while they themselves asked other questions than before. Nicole, for instance, told she had an extensive conversation with someone about why he gained so much weight, while she usually asked patients only about their current dietary and exercise behaviour. Ellen experienced a patient that used to be stiff to talk more freely during consultations.

In addition, Nicole mentioned she became more interested in certain topics, because she heard much more about her patients’ lifeworld experiences. An example of this was bariatric surgery, something several of her patients underwent, were on a waiting list for or thought about. One of her patients had such a surgery the week before the final walk:

Nicole tells me everything went well; he sent her a WhatsApp message after surgery. He also texted that his nurse over there did not know how to deal with his high blood glucose levels. Nicole answered him by texting this was normal after surgery and should get better in time. He sent back a message with a thank you from the other nurse. Nicole says another patient had bariatrics recently. (…) She also talked about it with yet another one, but he does not dare to at the moment. She plans to relate these three to each other, so they can exchange experiences. [location N, final walk]

This excerpt demonstrates both new and creative care practices, more informal connections between the nurse and her patients and another example of the organization of shared care. It illustrates how lifeworld experiences entered and impacted care relations.

## 4 DISCUSSION

This study focussed on care practices of two nurses working in a new context; instead of protocol‐based consultation room care, they provided 20 weeks of PA care outside by offering weekly walking sessions for people with type 2 diabetes. The organization of these walks was facilitated, but without a fixed script; how to offer this care was left to the nurses—illustrated by differences in the two approaches. Furthermore, our focus on system and lifeworld dynamics provided detailed insights into innovations in PA care practices and especially the impact on relationships between those involved.

Overall, underlying the four main themes found in this study were two important processes of developments in relations with and among participating patients, namely in nurse‐patient relationships and shared care. The aim of the discussion is to reflect on the value of these developments as well as on new questions that arise. Finally, these insights are used to reflect on implications for nursing responsibilities in the provision of PA care. Interestingly, although the starting point of the project was to help patients to become more active, the implications seemed to stretch beyond PA care and impact the provision of diabetes care more generally.

### 4.1 Nurse–patient relationships

Nurse–patient relationships are found to be shaped by the care context (Wiechula et al., 2016). The context in this particular study seemed to be more person‐centred instead of patient‐centred (Zhao, Gao, Wang, Liu, & Hao, 2016), with a focus shifting from illness towards walking together and creating opportunities to exchange more extended narratives about life and illness (Frank, 2013) than the consultation room permits. Moreover, since nurses and patients saw each other weekly for a longer period, it offered an opportunity for what Zhao et al. (2016) call 'continuous care'. As a result, they got to know each other better—beyond the patient aspects.

Deeper insights into the patients’ lifeworld experiences might contribute to a better professional understanding of patients’ PA behaviour, including their difficulties in becoming more active (Stuij, 2018; cf. Hinder & Greenhalgh, 2012). Furthermore, this might result in the development of a ‘common story’ about health enhancement that requires mutual commitment of both patient and nurse (Strandås & Bondas, 2018). In line with this, individual patient responsibility for PA behaviour became a form of shared responsibility because of the mutual commitment of everyone to this project.

In addition, the exchange between nurses and patients became more mutual during the project, as the nurses also shared information about themselves. Although the evidence is not unequivocal, this 'self‐disclosure' has the potential to enhance relationships with patients (Arroll & Allen, 2015). However, it might also create new professional dilemmas, especially related to keeping a 'professional distance' (Hem & Heggen, 2003) and the nurses’ own boundaries between their professional and personal life. For instance, they might face questions about what to share and whether they want to do this at all. Although the nurses in this study did not seem to face tensions related to this, it might also result in uncomfortable feelings.

### 4.2 Shared care

An important new feature of this project was offering care to a group of people. This brought new issues about how to care, for example, in dealing with private matters or caring for both quick and slow walkers. These questions were handled in practice. Moreover, group care brought new dynamics and offered the opportunity of shared care since patients came to take care of each other, as a form of social discipline (Huijer, 2013). This was also something the nurses learned to organize, for instance by connecting specific patients to each other. In this way, attention is refocused to what patients can learn from each other instead of from a nurse (Horstman & Houtepen, 2008).

However, care was not shared by everyone as a part of the patients quit during the project. Their reasons might be several, but, at a minimum, a fit with the walking project, the approach of the professional and preferably the pace and personalities of the others. This is something that cannot be controlled and thus, meet everyone's needs, as illustrated by the results. This creates new possible dilemma's, for instance, about how much extra effort to invest or when to let go of patients, especially when extra efforts require spare time. This might be even more complicated when behaviour change becomes a shared responsibility instead of an individual responsibility. In addition, colleagues of the organizing nurses were difficult to include in the project, especially the doctors, despite the efforts taken. Their reasons might also be several, although it illustrates a system–lifeworld hierarchy that was not countered, at least not at these two locations.

### 4.3 Reflections on nursing responsibilities in the provision of PA care

Given the difficulties of healthcare professionals in PA promotion practices (Hébert et al., 2012; Huijg et al., 2015; Stuij, 2018), PA care might benefit from more lifeworld‐led forms of care (Dahlberg, Todres, & Galvin, 2009). Although the current study did not aim to measure effects of the project on actual PA behaviour, the findings offer a starting point for a reflection on nursing responsibilities in the provision of (PA) care.

Overall, this project seemed to have ‘blurred’ the boundaries of both nursing roles and care provision, by literally stepping outside the consultation room and leaving regular protocols behind. This was underlined by the professionals feeling ‘outside the patient mode’ during the walks. This different approach seemed to help nurses to enrich their generalized evidence‐based knowledge of benefits of PA for their patients—most valued in the system world—with the concrete, local knowledge from their patients’ lifeworld, more than they normally acquired inside their consultation room. This type of knowledge is considered a prerequisite for good care (Hamington, 2018) and might contribute to the nurses’ tacit know‐how (Salter & Kothari, 2016) in providing PA care.

Providing practical and social self‐management support is found to be important in type 2 diabetes care (Oftedal et al., 2010).Taking patients by the hand might contribute to this, as it fosters the development of care attuned to personal needs and wishes through attentiveness and presence. As such, it fits with an ethics of care that directs nurses’ attention to responsiveness in relationships (Abma & Baur, 2014). In this process, the nurses’ responsibility shifted from expert education towards facilitation (Jacobs, 2011) of PA possibilities and shared care. Furthermore, participation in this project might offer nurses a possibility to reflect on their own role and caring responsibilities in providing PA care and counselling (Jacobs, 2011).

Participation of the two nurses in this project showed their intrinsic motivation to improve PA care and offered them a rich learning experience. However, being more outside the system world might also offer new risks and dilemmas, for instance, about maintaining a professional distance and the investment of time. Inside the consultation room, time is limited, or at least more or less. During this project, the requested investment of time was not limited in a similar way. Although the nurses probably did not know beforehand how much time was needed, it was not possible to (partly) withdraw during the project, because of their commitment towards patients. Especially the investment of their own spare time caused a dilemma between their own interest and solidarity with their patients (Van den Ende & Kunneman, 2008).

Therefore, professional innovations as illustrated in this study, also require organizational and policy adaptations (Stephen et al., 2018) to care for the nurses themselves. For instance, they need sufficient support to carry out the organizational efforts for such a project. Moreover, this project raises questions about the provision of PA care as part of self‐management support. For instance, whether such a project should be part of nursing care at all. Given the results presented in this study, PA care might benefit from a more playful handling of the system and lifeworld balance and further exploration of creative elements in (PA) care for people with type 2 diabetes.

### 4.4 Limitations

A limitation of this study is that we did not talk to patients who quit or doctors who did not take part. Their insights might have added extra information, for instance, about whether doctors were unable or unwilling to join. Furthermore, the perspective of the patients was hardly included in this study. Finally, the two cases in this study were not indicative for all locations where the project was carried out. For instance, the learning process could have turned out differently, or failed.

## 5 CONCLUSION

This study provided a detailed examination of care practices of nurses organizing weekly walks for people with type 2 diabetes. The focus on system and lifeworld dynamics offered in‐depth insights into the potential of these new practices and especially revealed relational developments during the project. Stepping outside the consultation room seems to offer more space for patients’ lifeworld narratives and contribute to more continuous and person‐centred care. However, it also raises new questions about the provision of PA care and related nursing responsibilities. These questions need to be further discussed, for instance, by policy makers, healthcare professionals and researchers, to further develop the provision of 'good' PA care for people with type 2 diabetes.

## CONFLICT OF INTEREST

No conflict of interest has been declared by the authors.

## AUTHOR CONTRIBUTIONS

MS, AE, and TA made substantial contributions to conception and design, or acquisition of data, or analysis and interpretation of data; MS, AE and TA were involved in drafting the manuscript or revising it critically for important intellectual content; MS, AE and TA gave final approval of the version to be published. Each author participated sufficiently in the work and was responsible for appropriate portions of the content; MS, AE and TA agreed to be accountable for all aspects of the work in ensuring that questions related to the accuracy or integrity of any part of the work are appropriately investigated and resolved.
